# Sulforaphane, a Dietary Isothiocyanate, Induces G_2_/M Arrest in Cervical Cancer Cells through CyclinB1 Downregulation and GADD45β/CDC2 Association

**DOI:** 10.3390/ijms17091530

**Published:** 2016-09-12

**Authors:** Ya-Min Cheng, Ching-Chou Tsai, Yi-Chiang Hsu

**Affiliations:** 1 Department of Obstetrics and Gynecology, National Cheng Kung University Hospital, College of Medicine, National Cheng Kung University, Tainan 70403, Taiwan; 2 Institute of Clinical Medicine, College of Medicine, National Cheng Kung University, Tainan 70403, Taiwan; 3 Division of Gynecologic Oncology, Department of Obstetrics and Gynecology, Chang Gung Memorial Hospital and Chang Gung University College of Medicine, Kaohsiung 83301, Taiwan; nick@cgmh.org.tw; 4 Department of Obstetrics and Gynecology, Chang Gung Memorial Hospital, Chang Gung University College of Medicine, Chiayi 61333, Taiwan; 5 Graduate Institute of Medical Science, College of Health Sciences, Chang Jung Christian University, Tainan 71101, Taiwan; 6 Bachelor Degree Program of Medical Sciences Industry, College of Health Sciences, Chang Jung Christian University, Tainan 71101, Taiwan

**Keywords:** sulforaphane, cervical cancer cells, G_2_/M arrest, cyclin B1/CDC2, growth arrest and DNA damage 45 (GADD45)

## Abstract

Globally, cervical cancer is the most common malignancy affecting women. The main treatment methods for this type of cancer include conization or hysterectomy procedures. Sulforaphane (SFN) is a natural, compound-based drug derived from dietary isothiocyanates which has previously been shown to possess potent anti-tumor and chemopreventive effects against several types of cancer. The present study investigated the effects of SFN on anti-proliferation and G_2_/M phase cell cycle arrest in cervical cancer cell lines (Cx, CxWJ, and HeLa). We found that cytotoxicity is associated with an accumulation of cells in the G_2_/M phases of the cell-cycle. Treatment with SFN led to cell cycle arrest as well as the down-regulation of Cyclin B1 expression, but not of CDC2 expression. In addition, the effects of GADD45β gene activation in cell cycle arrest increase proportionally with the dose of SFN; however, mitotic delay and the inhibition of proliferation both depend on the dosage of SFN used to treat cancer cells. These results indicate that SFN may delay the development of cancer by arresting cell growth in the G_2_/M phase via down-regulation of Cyclin B1 gene expression, dissociation of the cyclin B1/CDC2 complex, and up-regulation of GADD45β proteins.

## 1. Introduction

Cervical cancer is the sixth most common cancerous malignancy among women in Taiwan [1]. Worldwide, cervical cancer is also one of the most common cancers affecting women and is a leading cause of gynecological death [2,3]. The majority of women diagnosed with this cancer exhibit advanced, widely disseminated malignancies, and have a poor survival rate [4]. Infection with human papillomavirus (HPV) is believed to play a particularly important role in the initiation of cancer formation [5]. Nonetheless, only a small fraction of females infected with HPV develop cancer [6], indicating that other factors also contribute to the progression of carcinogenesis, including genetic predisposition and environmental influences [7].

Many studies indicate that the consumption of cruciferous vegetables is positively correlated with decreased incidence of some tumors, including those of the prostate, cervical, ovarian, lung, and gastrointestinal tract [8]. Dietary isothiocyanates (ITCs) are glucosinolate degradation products that occur naturally in certain vegetables and plants shown to possess chemopreventative properties [9,10] and biological activity against carcinogenesis [11]. Sulforaphane ((1-isothiocyanato-(4*R*,*S*)-(methylsulfinyl)butane); SFN), a potent chemopreventive agent, is an ITC compound that occurs as a precursor glucosinolate in cruciferous vegetables, such as brussel sprouts, cauliflower, and broccoli [12]. Due to its beneficial pharmacological effects, including antitumor, anti-inflammatory and antioxidant effects, interest in SFN has grown in recent years [13]. SFN is also reported to be a potent scavenger of reactive oxygen species (ROS) and is able to suppress specific inflammatory factors [14]. In addition to inhibiting cell proliferation, SFN can increase apoptosis [15], induce the accumulation of phase-II detoxification enzymes, and inhibit cyclooxygenase 2 (COX-2) [16] and matrix metalloproteinase (MMP), the effect on protein kinases and others [17]. However, whether SFN is able to inhibit the growth of cervical cancer cells growth remains unknown, and the mechanisms underlying the anti-cancer effects of SFN have not yet been identified.

This present study investigated whether SFN contributes to anti-proliferation and cell cycle arrest in cervical cancer cell lines. We believe that results from these experiments provide scientific and technological support to facilitate the further development of treatment therapies for cervical cancer.

## 2. Results and Discussion

### 2.1. SFN Inhibits Cell Survival in Cervical Cancer Cell Lines

**Figure 1:**
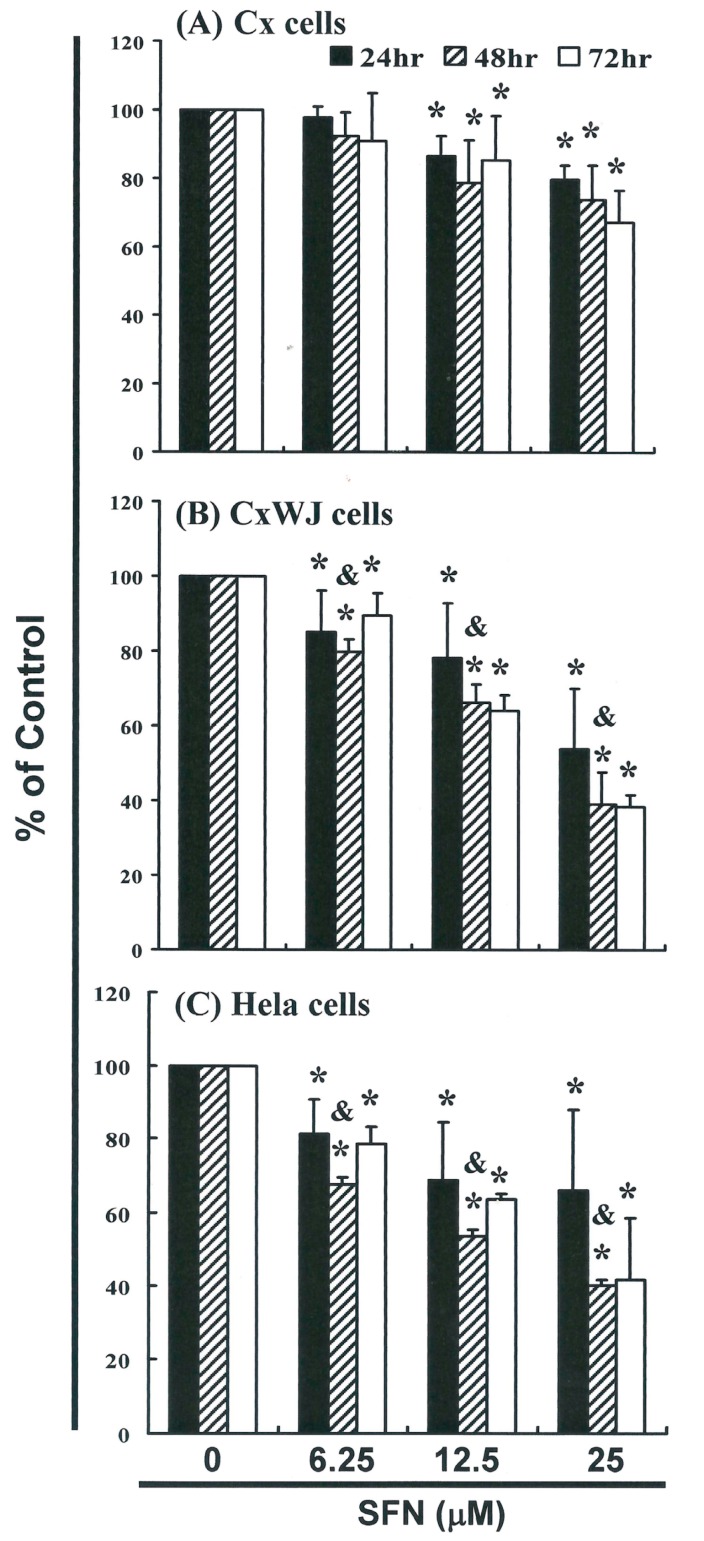
SFN inhibits the survival of cervical (**A**) Cx; (**B**) CxWJ and (**C**) HeLa cancer cell lines, thereby inhibiting proliferation. Cancer cell lines were treated with increasing doses of SFN (0, 6.25, 12.5 and 25 μM) for 24 to 72 h, and cell survival was quantified using the MTT method. Results were expressed as a percentage of the control, which was considered to be 100%. All data are reported as the mean (±SEM) of at least three separate experiments. Statistical analysis was performed using a *t*-test, with differences considered significant at a level of *p* < 0.05. (The * symbol indicates that results were significant versus the control group, and the & symbol indicates that results were significant versus the 24 h group).

To explore this anti-tumor activity, we conducted an in vitro study that involved subjecting Cx, CxWJ, and HeLa cell lines to increasing dosages of SFN (0, 6.25, 12.5, and 25 μM) for 24 to 72 hours. The proliferation of SFN-treated cancer cell lines was measured using the MTT method (Figure 1). Results of this investigation indicate that treatment with SFN decreased the survival and proliferation of Cx (Figure 1A), CxWJ (Figure 1B), and HeLa (Figure 1C) cells in a dose—(Cx: *y* = −7.2569*x* + 109.1, *R*^2^ = 0.9466; CxWJ: *y* = −14.511*x* + 115.59, *R*^2^ = 0.9523; HeLa: *y* = −11.356*x* + 107.49, *R*^2^ = 0.9085) and time-dependent manner.

### 2.2. Non-SFN-Induced Apoptosis/Necrosis of Cx, CxWj and HeLa Cell Lines

To identify the role played by SFN in the apoptosis/necrosis of cervical cancer lines, we stained cells with Annexin V-FITC and propidium iodide, which led to apoptosis following 4 h of SFN exposure. The percentage of apoptotic cells was assessed using flow cytometric analysis (Figure S1A–C).

**Figure 2:**
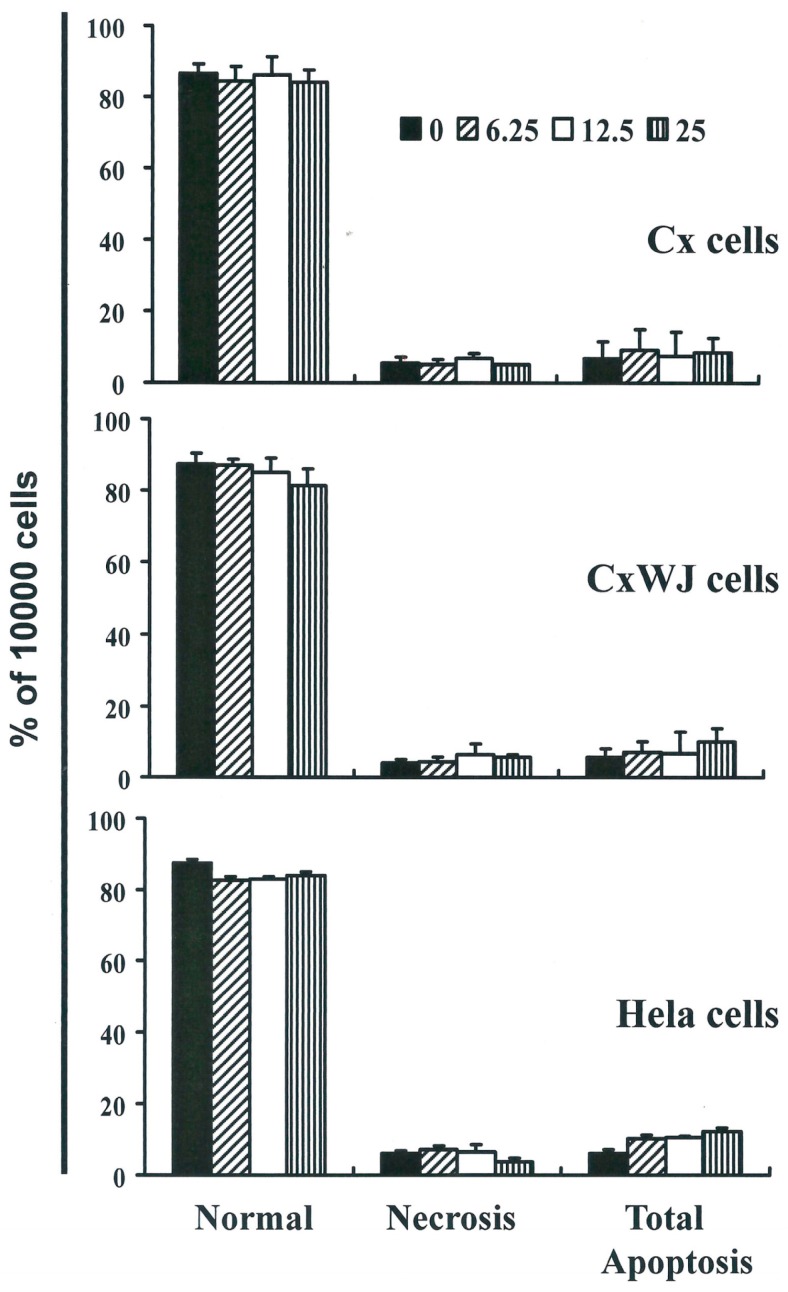
Total apoptosis and necrosis in cervical cancer cell lines after 4 h of incubation with SFN (0, 6.25, 12.5, and 25 μM). Results are expressed as a percentage of control group cells, necrosis, and total number of apoptotic cells (including both early and late apoptosis).

A dot-plot of Annexin V-FITC fluorescence versus PI fluorescence indicates that compared with untreated cells, SFN treated cells underwent a non-significant increase in apoptosis. Indeed, no significant increases were observed in the percentage of cells undergoing necrosis or apoptosis (Figure 2) but caspase 3 activation at SFN concentrations of 6.25 to 25 μM in CxWJ (*y* = 6.6047*x* − 5.59, *R*^2^ = 0.9503) and HeLa cells (*y* = 10.08*x* − 3.5683, *R*^2^ = 0.9216) (Figure S2A,C). In addition, we did not observe any significant changes in the concentrations of pro-caspase-3, -8, -9 and AIF in SFN-treated HeLa cells (Figure S3).

Assessment of changes in mitochondrial membrane potential in SFN-treated cells. The loss of mitochondrial membrane potential is a hallmark of apoptosis. This loss is an early event that coincides with caspase activation. In non-apoptotic cells, JC-1 exists as a monomer in the cytosol (green) and as aggregates in the mitochondria, where it appears red. In apoptotic and necrotic cells, JC-1 exists in monomeric form and stains the cytosol green. Figure S2B shows typical FL-1/FL-2 dot plots for JC-1 staining of CxWJ and HeLa cells with and without apoptosis. CxWJ and HeLa cells that were not treated with SFN did not undergo apoptosis and thus contain red fluorescing J-aggregates. Conversely, the green fluorescing monomers shown in the lower part of the figure indicate apoptotic cells. Figure S2D shows the percentages of apoptotic cells (as analyzed by flow cytometry) in different SFN-treated groups (CxWJ: *y* = 3.7483*x* − 1.4017, *R*^2^ = 0.8597 and HeLa: *y* = 0.9677*x* + 5.5033, *R*^2^ = 0.2605). Taken together, these observations suggest that SFN reduced the mitochondrial membrane potential of cervical cancer cell lines, albeit in a non-significant way.

Results summarized in Figures S1–S3 nonetheless indicate that SFN may mediate the survival of cervical cancer cell lines. We therefore hypothesize that the proliferation of these cells was inhibited by a mechanism that does not involve apoptosis/necrosis.

### 2.3. SFN Induced the Accumulation of Cells in the G_2_/M Phase

**Figure 3:**
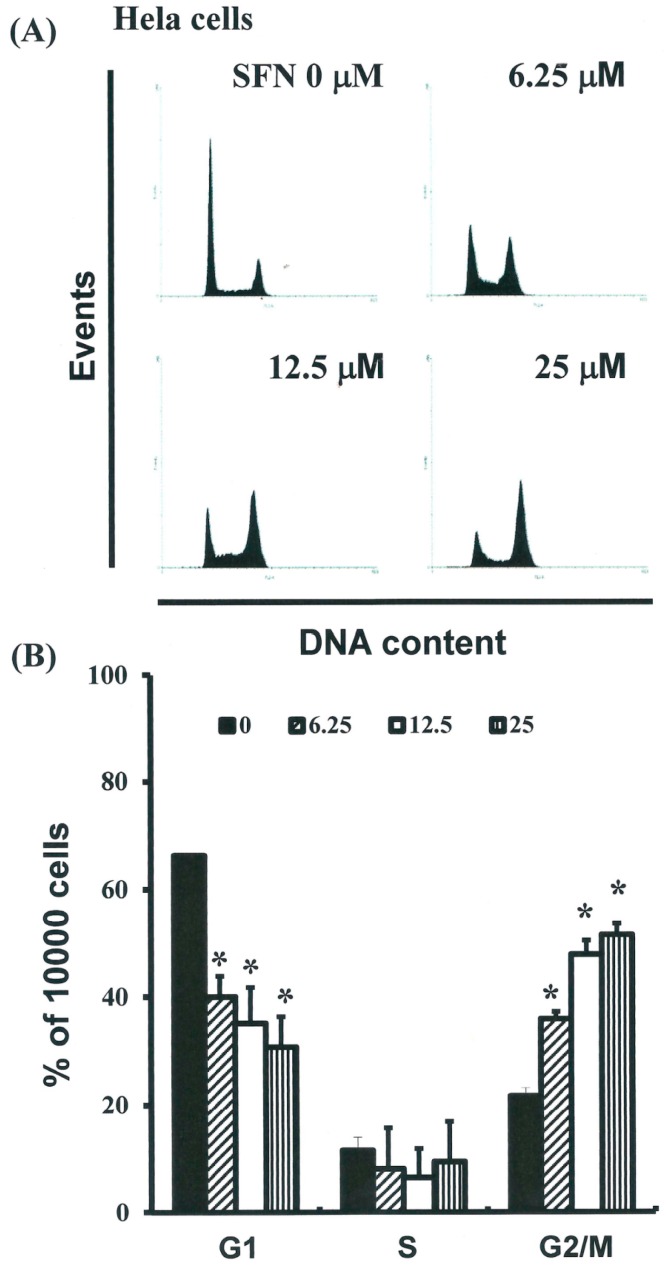
Influence of SFN on cell cycle progression/distribution in HeLa cells: (**A**) Cell cycle analysis of HeLa cells cultured with SFN for 24 h; (**B**) SFN induced an increase in G_2_/M phase cells and a decrease in G1 phase cells (results are expressed as percentages). The * symbol in each group of bars indicates that differences resulting from treatment with SFN 0 µM were statistically significant at *p* < 0.05.

The cell-cycle distribution of SFN-treated cells was analyzed by flow cytometry. For this, cells were exposed to SFN for 24 h prior to processing and analysis. Exposure to SFN led to an increase in the number of G_2_/M phase cells (*y* = 10.17*x* + 13.875, *R*^2^ = 0.9456) (Figure 3A) as well as a simultaneous decrease in G_1_ phase cells (* *p* < 0.05 vs. SFN 0 μM) (*y* = −11.145*x* + 70.935, *R*^2^ = 0.8129). These results imply that the HeLa cells underwent cell cycle arrest.

### 2.4. Effects of SFN on the Mitotic Index

**Figure 4:**
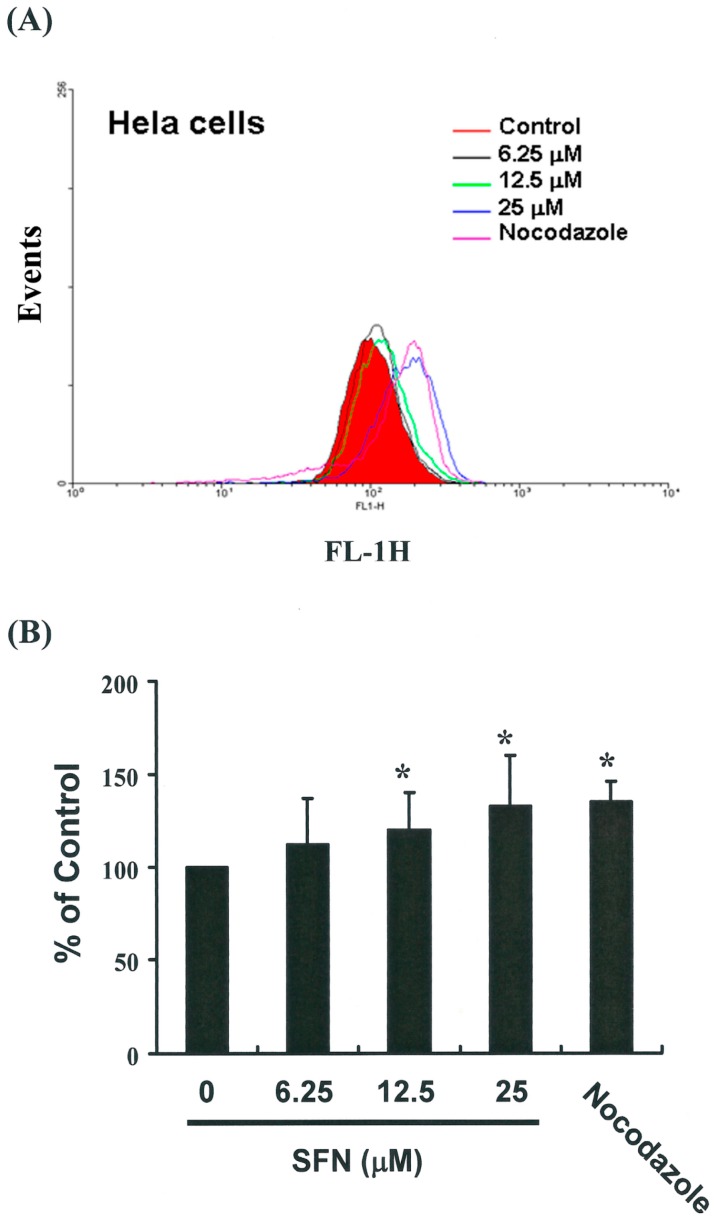
Influence of SFN on MPM-2 (anti-phospho-Ser/Thr-Pro) expression in untreated and treated cancer cells. MPM-2 is an antibody that recognizes proteins which are only phosphorylated during mitosis. Cells were dually stained using propidium iodide to analyze DNA content, and protein expression was quantified by flow cytometry. As a positive control, separate groups of cells were treated with nocodazole for 24 h (15 μg/mL), an anti-fungal agent known to induce metaphase arrest. (**A**) MPM-2 expression (gated cells) was measured by flow cytometry following treatment with SFN for 24 h; (**B**) SFN enhanced the level of MPM-2 in HeLa cells. The * symbol in each group of bars indicates that differences resulting from treatment with SFN 0 μM were statistically significant at *p* < 0.05.

To distinguish G2 arrest from mitotic arrest, we employed the marker MPM-2 (anti-phospho-Ser/Thr-Pro), which is capable of recognizing proteins whose epitopes are exclusively phosphorylated during mitosis, specifically, from early prophase to metaphase [14]. MPM-2 is commonly used to identify mitotic disturbance. To provide a positive control, we treated separate groups of HeLa cells with nocodazole (15 μg/mL), an inducer of metaphase arrest [15], for 24 h. This led to the synchronization of entire cell populations in the G_2_/M phase and an increase in MPM-2 labeling (Figure 4A). All cells treated with SFN showed elevated MPM-2 levels compared with the control group (12% to 32% for HeLa cells treated with SFN) (*y* = 10.543*x* + 89.882, *R*^2^ = 0.9907) (Figure 4B). However, MPM-2 staining was not as strong as that achieved with nocodazole (35%).

This is likely because cells stained with MPM-2 were in various stages of mitosis, some of which could not be identified using this early prophase marker (i.e., cells which had accumulated in the G_2_/M phase were not necessarily marked). Thus, our experiments involving MPM-2 staining may have underestimated the degree of mitotic disturbance.

### 2.5. G_2_/M Phase Arrest in SFN-Treated Cells via Cyclin B1 Down-Regulation and GADD45β Up-Regulation

**Figure 5:**
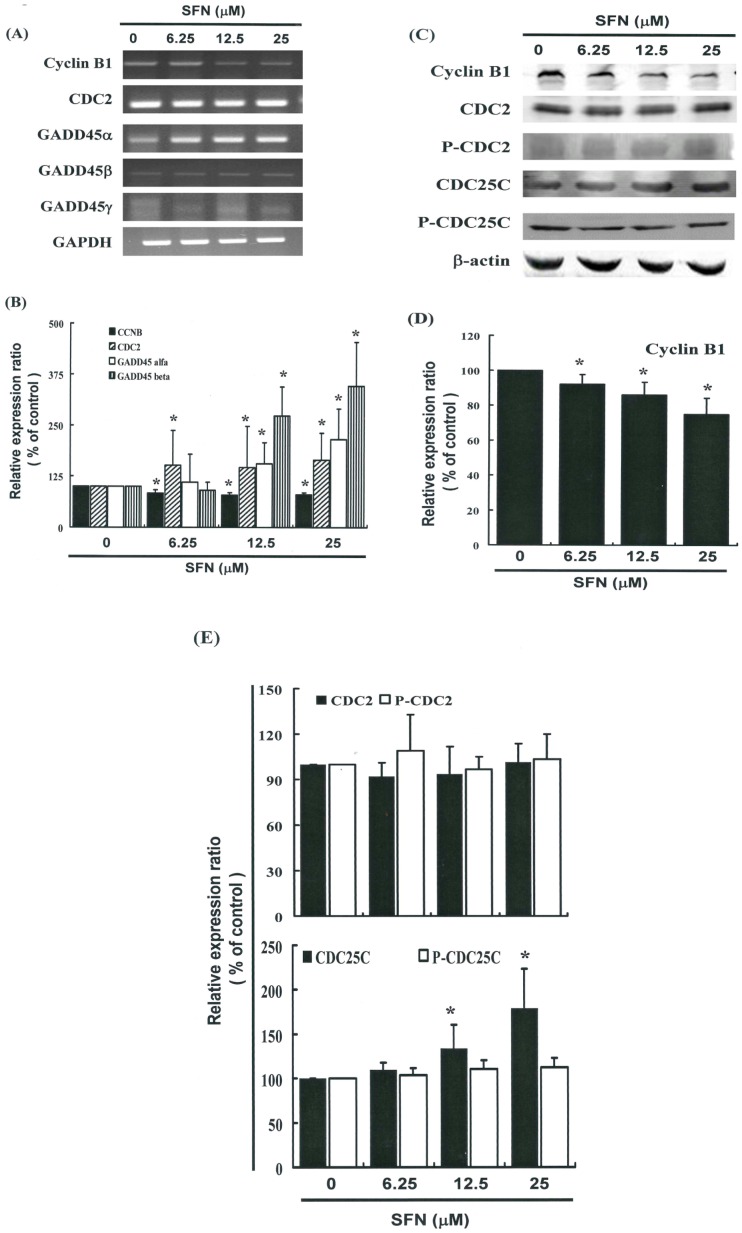
SFN represses cyclinB1 and up-regulates GADD45β gene expression in HeLa cells. The cells were treated with SFN (0, 6.25, 12.5, and 25 μM) for 24 hours, and gene expression was subsequently detected by RT-PCR, quantitative RT-PCR (qPCR) and Western blot analysis. (**A**) RT-PCR results for cyclin B1, CDC2 and, GADD45 family mRNAs in HeLa cells following exposure to SFN; (**B**) The panels show qPCR analysis of mRNA expression in cyclin B1, CDC2, GADD45-α, -β and -γ; (**C**) Representative blot from 3 independent experiments; (**D**,**E**) Quantification of band intensities. All data is reported as the mean (±SEM) of at least three separate experiments. Statistical analysis was performed using a *t*-test, with differences considered significant at a level of * *p* < 0.05 versus the 0 μM control group.

**Figure 6:**
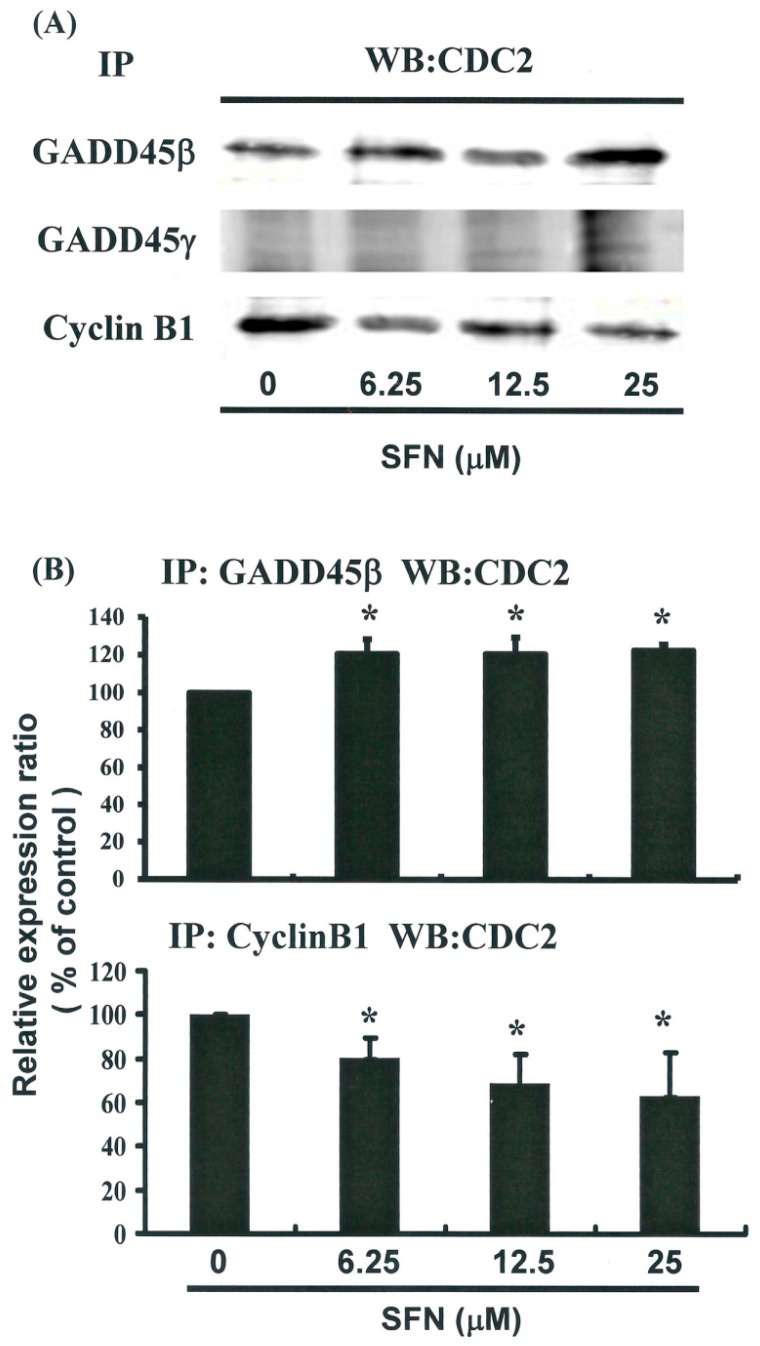
Influence of SFN on the association between CDC2 and GADD45-β/-γ in HeLa cells: (**A**) Co-IP of Cyclin B1, GADD45-β, and GADD45-γ with CDC2 in HeLa cells which had been treated with SFN for 24 h; (**B**) Quantification of band intensities. All data were reported is the mean (±SEM) of at least three separate experiments. Statistical analysis was performed using a *t*-test, with differences considered significant at a level of * *p* < 0.05 versus the 0 μM control group.

Figure 5A illustrates the expression of cyclin B1 and GADD45 family mRNA genes. Figure 5C provides immunoblots of cellular proteins from HeLa cells treated with SFN. Gene expression and Western blot analysis revealed that cyclin B1 decreased following incubation with SFN (Figure 5A–C). Western blotting (Figure 5C) also revealed that SFN treatment was associated with a decrease in cyclin B1 protein expression in HeLa cells. Co-IP further demonstrated that the GADD45β/CDC2 complex had formed (Figure 6A,B) (*y* = 6.8638*x* + 98.995, *R*^2^ = 0.6716), but the GADD45β/Cyclin B1 complex had not (data not shown). Expression of Cyclin B1 proteins was quantified by measuring relative band intensities (*y* = −6.7396*x* + 102, *R*^2^ = 0.733) (Figure 5C). This analysis further confirmed that cyclin B1/CDC2 complex levels were significantly lower in cells that had been incubated with SFN (Figure 6A,B) (*y* = −12.179*x* + 108.55, *R*^2^ = 0.9358).

### 2.6. Effects of SFN on the CDC2 and CDC25C in HeLa Cells

Figure 5C illustrates the expression of p-CDC2, CDC2, p-CDC25C, and CDC25C proteins and also provides immunoblots of cellular proteins from SFN treated HeLa cells. Here, protein expression was quantified by measuring relative band intensities. Western blot analysis revealed that the CDC25C/p-CDC25C ratio increased (*y* = 0.1898*x* + 0.7341, *R*^2^ = 0.8615) following incubation with SFN (Figure 5E); however, the ratio of p-CDC2/CDC2 did not (*y* = −0.0092*x* + 1.0817, *R*^2^ = 0.0204).

These findings suggest that the mechanism by which SFN mediates the tumorigenicity of cervical cancer cells involves the regulation of cellular cyclin B1 and GADD45β levels. This in turn indicates that the induction of cell cycle arrest in the G_2_/M phase involves common molecular pathways. Results from qPCR analysis (Figure 5B) were further validated using microarray analysis, which revealed substantial down-regulation of cyclin B1 as well as notable up-regulation of the GADD45β and the GADD45β/CDC2 complexes in cervical cancer cells following exposure to SFN (Figure 6).

Sulforaphane, a naturally occurring alkyl isothiocyanate, has been shown to possess more potent cytotoxicity against cancer cells than other synthetic isothiocyanate analogues. The most common type of cell death associated with SFN treatment appears to be apoptosis, and many studies have attempted to elucidate the mechanism underlying the anticancer activities of this compound [18,19]. 

In the current investigation, SFN demonstrated anticancer activity as well as the ability to induce a delay in mitosis. We found that treating cervical cancer cell lines with SFN led not only to the down-regulation of Cyclin B1 protein expression, but resulted in the dephosphorylation of CDC25C as well. CDC2 proteins were shown to bind with GADD45β, which mediated both the G1 and G_2_/M phases of the cell cycle [20]. Previous studies have also provided evidence which suggests that the binding of CDC2 with GADD45β and genes from the Gadd45 family is important in inducing anti-cancer immune responses [21,22].

CDC2 tyrosine phosphorylation is maintained during cell cycle arrest, and phosphorylation of CDC2 on Thr14 and Thr 15 inhibits CDC2 activity [21]. In HeLa cells, mitotic delays induced by SFN do not necessarily result from tyrosine phosphorylation (Figure 5C,E), but rather from the down-regulation of Cyclin B1 gene expression (Figure 5A–D), the up-regulation of GADD45β gene expression, and the formation of the GADD45β/CDC2 complex (Figure 6).

Results collected in this series of studies provide experimental evidence to support the contention that SFN can irreversibly arrest the growth of cervical cancer cells. The results of our mechanistic analysis lead us to conclude that both the inhibition of proliferation and the induction of cell cycle arrest are highly dependent upon SFN accumulation in cancer cells.

## 3. Experimental Section

### 3.1. Materials

Sulforaphane, MTT (3-(4,5-dimethylthiazol-2-yl)-2,5-diphenyltetrazolium bromide) and DMSO (dimethyl sulfoxide) were obtained from Sigma (St. Louis, MO, USA). Cell culture medium (MEM medium), phosphate-buffered saline (PBS), antibiotics, sodium pyruvate, trypsin, and fetal bovine serum were purchased from Gibco, BRL (Grand Island, NY, USA). Polyvinylidene fluoride membrane (PVDF) (Merck Millipore, Billerica, MA, USA), and molecular weight markers were purchased from Bio Rad (Hercules, CA, USA). All other reagents and compounds were analytical grade.

### 3.2. Cells

HeLa cells were purchased from ATCC. Cx and CxWJ cell lines were obtained from patients admitted to the Department of Obstetrics and Gynecology at the National Cheng Kung University (NCKU) Medical Center [1,2]. HeLa cells were maintained on culture dishes in 90% (*v*/*v*) minimum essential medium Eagle with 2 mM l-glutamine and Earle’s BSS adjusted to contain 1.5 g/L sodium bicarbonate, 0.1 mM non-essential amino acid, and 1 mM sodium pyruvate with 10% (*v*/*v*) fetal bovine serum (FBS). Cell cultures were maintained in an incubator under an atmosphere of 5% CO_2_ at a temperature of 37 °C.

### 3.3. Cell Proliferation Assay

Cells were seeded into a 96-well culture plate at a density of 5000 cells/well and treated with 0, 6.25, 12.5, or 25 μM SFN, the SFN will complex with medium. The cells were then incubated at 37 °C for 24, 48, and 72 h, respectively. After the incubation periods (i.e., 24, 48, and 72 h) were complete, cells were treated with MTT dye (1 mg/mL per well) and allowed to sit for at least 4 h. We then stopped the reaction through the addition of DMSO and used a multi-well plate reader to measure optical densities at 540 nm. For this determination, the background absorbance of the medium in the absence of cells was subtracted. All samples were assayed in triplicate, and the mean for each experiment was calculated. Results are expressed as a percentage of that control, which was assumed to be 100%. All reported values are expressed as the mean (±SEM).

### 3.4. Measurement of Apoptosis

Cervical cancer cell lines were seeded in 6-well plates (Orange Scientific, Braine-l'Alleud, Belgium) and treated with SFN for four hours before being harvested. The cells were then re-centrifuged and resuspended/incubated in 1× annexin-binding buffer (the supernatant was discarded) before being treated with five μL of annexin V-FITC (BD Pharmingen, BD, San Diego, CA, USA) and one μL of 100 μg/mL PI working solution for 15 min. Following the incubation period, the stained cells were analyzed using flow cytometry (FACSCalibur, BD, USA) and open-source software WinMDI 2.9 (BD, San Diego, CA, USA).

### 3.5. Caspase 3 Activity Assay

Caspase activity was assessed using FITC rabbit anti-active caspase-3 (BD Pharmingen, USA). Cells were treated with 0, 6.25, 12.5, or 25 μM SFN for 24 h. Caspase activity was then analyzed using flow cytometry (FACSCalibur, BD, USA) and the open-source software WinMDI 2.9 (BD, USA).

### 3.6. Mitochondrial Membrane Potential (MMP)

Cell lines were first seeded in 6-well plates (Orange Scientific, E.U.) and treated with SFN for four hours. Following SFN treatment, JC-1 (25 μM) was added to the culture medium (500 µL/well) and the cells were incubated (37 °C) for 20 min prior to mitochondrial staining. For this, cells were washed twice with warm PBS before being fixed using 2% paraformaldehyde. The level of JC-1 was quantified using flow cytometry (BD FACScalibur, BD, USA), where mitochondria containing red JC-1 aggregates in healthy cells were detectable in the FL2 channel, and green JC-1 monomers in apoptotic cells were detectable in the FL1 channel.

### 3.7. Cell Cycle Analysis

To facilitate cell cycle analysis, we used the fluorescent nucleic acid dye propidium iodide (PI) to identify the proportion of cells in each of the three stages of interphase. For this, cells were treated with SFN for 24 h before being harvested and fixed in 1 mL cold 70% ethanol for at least 8 h at −20 °C. DNA was stained using PI/RNaseA solution, and DNA content was detected using flow cytometry. Data were analyzed using the open-source software WinMDI 2.9.

### 3.8. Mitotic Index Analysis

The mitotic index was assessed according to MPM-2 (anti-phospho-Ser/Thr-Pro) expression. Following treatment with SFN, cells were harvested and fixed in 70% ethanol overnight. Cells were then washed and suspended in 100 μL of IFA-Tx buffer (4% FCS, 150 nM NaCl, 10 nM HEPES, 0.1% sodium azide, 0.1% Triton X-100) with MPM-2 antibodies (1 μg/mL; Upstate Cell Signaling Solutions, Millipore, Watford, UK) at room temperature for 1 h. Subsequently, cells were washed and resuspended in IFA-Tx buffer with rabbit anti-mouse FITC-conjugated secondary antibodies (1:50; Serotec, Oxford, UK) for 1 h at room temperature in darkness. Finally, cells were washed and resuspended in 500 μL of PBS with 20 μg/mL of PI (Sigma) for 30 min in the dark. MPM-2 expression was analyzed using flow cytometry (FACSCalibur, BD, USA) the open-source software WinMDI 2.9 (BD, USA).

### 3.9. Western Blot Assay

A total of 50–100 μg of proteins were separated by 10% SDS-PAGE and transferred to PVDF membranes. Membranes were then blocked with blocking buffer (Odyddey, Lincoln, NE, USA) overnight before being incubated with anti-β-actin (Sigma-Aldrich, St. Louis, MO, USA), anti-caspase 3 (sc-7148), anti-caspase 8 (sc-6134), anti-caspase 9 (sc-7885), anti-AIF (sc-9417), anti-cyclin B1 (sc-752), anti-CDC2 (p34; sc-747), anti-p-CDC2 (p34 Tyr 15; sc-7989), anti-CDC25C (sc-13138), and anti-p-CDC25C (sc-12354) (Santa Cruz BioTechnology, Dallas, TX, USA) antibodies for 1.5~2 h. Blots were subsequently washed and (1) incubated with a second antibody (IRDye Li-COR, USA) or (2) conjugated with horseradish peroxidase (HRP) at a 1/20,000 dilution for 30 min. Antigens were then visualized using a near infrared imaging system (Odyssey LI-COR, Lincoln, NE, USA) or a chemiluminescence detection kit (ECL; Amersham Corp., Arlington Heights, IL, USA). Data were analyzed using Odyssey 2.1 software (Odyssey LI-COR).

### 3.10. Co-Immunoprecipitation (Co-IP)

Co-IP is an effective means of quantifying protein–protein interactions in cells. Briefly, after being incubated at room temperature overnight, 500 mg of cellular proteins were labeled using anti-CyclinB1 (sc-752), GADD45β (sc-33172) (Santa Cruz BioTechnology) and GADD45γ (STJ93197) (St John’s Lab, London, UK). The protein-antibody immunoprecipitates were collected using protein A/G plus-agarose beads (SC-2003 Santa Cruz BioTechnology). Following the final wash, the samples were boiled and centrifuged to transform the agarose beads into pellets. Finally, we conducted Western blot analysis of CDC2 proteins in the supernatant. Antigens were visualized using a near infrared imaging system (Odyssey LI-COR), and data were analyzed using Odyssey 2.1 software.

### 3.11. Real-Time PCR

A reverse transcriptase system (Promega, Southampton, UK) was used to synthesize cDNA from 1 μg of total RNA, and between 2 and 4 μL of cDNA was used for PCR analysis. PCR (50 μL) reactions were performed using 1 unit of Dynazyme II (Flowgen, Lichfield, UK) and 100 ng of each primer [16]. Thermal cycling was conducted through 35 cycles at the following temperatures and durations using a Progene thermal cycler (Cambridge, UK): 98 °C for 10 s, 66 °C for 30 s, and 72 °C for 1 min. At the end of 35 cycles, a final extension of 72 °C was performed. To ensure specificity, primers used to amplify the target genes in order to check them against all other gene sequences. PCR reactions were analyzed using 1.5% agarose/TAE minigels and stained using 0.5 μg/mL ethidium bromide. Gels were visualized using an Apligene UV CCD camera system.

### 3.12. Quantitative Real-Time PCR

Quantitative real-time PCR (qRT-PCR) was performed using approximately 200 ng of SYBR Green PCR MasterMix in an ABI 7300 system (Applied Biosystems, Foster City, CA, USA). Forty cycles of PCR were conducted at the following temperatures and durations: 95 °C for 120 s, 60 °C for 30 s, and 72 °C for 30 s. Sample cells from three plates were run in duplicate and the threshold suggested by the software was adopted for *C*_t_ calculations. To normalize readings, we used *C*_t_ values obtained at 18 s as internal controls for each run, and were thus able to calculate a delta *C*_t_ value for each gene. All protocols we adopted in performing qRT-PCR were in accordance with manufacturer’s instructions.

### 3.13. Statistical Analysis

A *t*-test or one-way ANOVA with post-hoc test were used *r* the analysis of results, with a significance level of *p* < 0.05. All data are reported as the mean (±SEM) of at least three separate experiments. For each standard curve, a regression equation can be derived and used for calculating SFN concentration (*x* axis) with response (*y* axis).

## 4. Conclusions

In conclusion, this study provides novel evidence to demonstrate that SFN is an effective inhibitor of cervical cancer. The role that SFN may plays in the inhibition of tumor growth was highlighted by the delay of mitosis through the down-regulation of cyclin B1 and the dissociation of the cyclin B1/CDC2 complex by GADD45β in cervical cancer cell lines. SFN is also reported to inhibit cell proliferation by apoptosis [14,15] and chemoprevention [23]. Taken together, these findings suggest that SFN can be applied as an anti-tumor agent in cervical cancer chemotherapies.

## Supplementary Materials

Supplementary materials can be found at www.mdpi.com/1422-0067/17/9/1530/s1.
