# Supplementary material for: Sulforaphane, a Dietary Isothiocyanate, Induces G2/M Arrest in Cervical Cancer Cells through CyclinB1 Downregulation and GADD45β/CDC2 Association
Source: Int J Mol Sci. 2016 Sep 12;17(9):1530. doi: 10.3390/ijms17091530 (PMC5037805; doi:10.3390/ijms17091530)
Supplement: Supplementary file 1 [file ijms-17-01530-s001.pdf]

Supplementary Materials: Sulforaphane, a Dietary Isothiocyanate, Induces G_2/M Arrest in Cervical Cancer Cells through CyclinB1 Downregulation and GADD45β/CDC2 Association

Ya-Min Cheng, Ching-Chou Tsai and Yi-Chiang Hsu

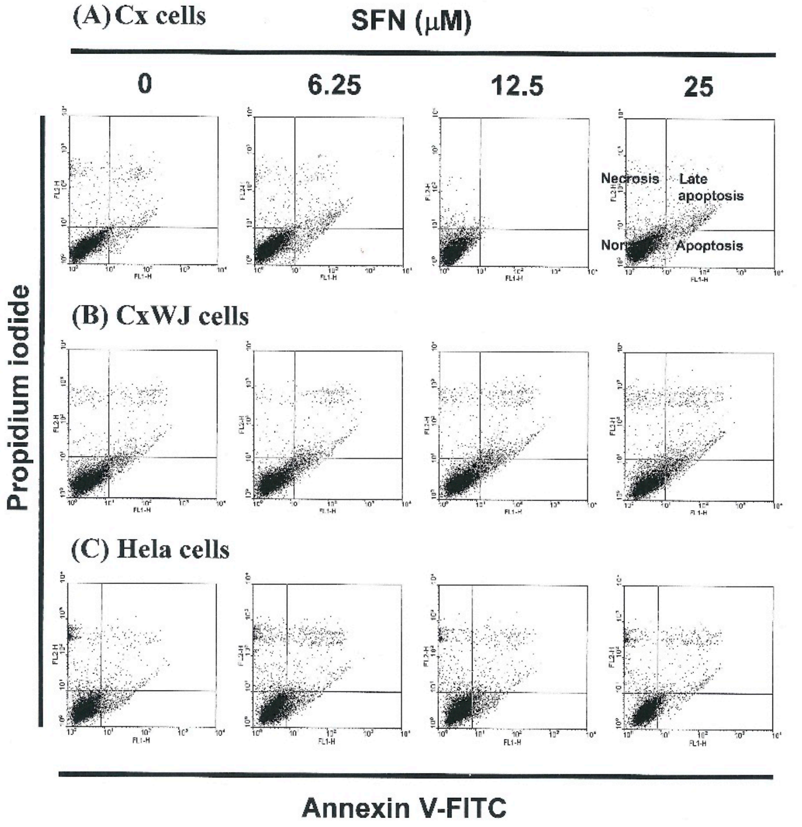

Figure S1. Influence of SFN on apoptosis/necrosis in cervical cancer cell lines (**A**) Cx, (**B**) CxWJ and (**C**) HeLa. Total apoptosis and necrosis in cervical cancer cell lines after 4 hours of incubation with SFN.

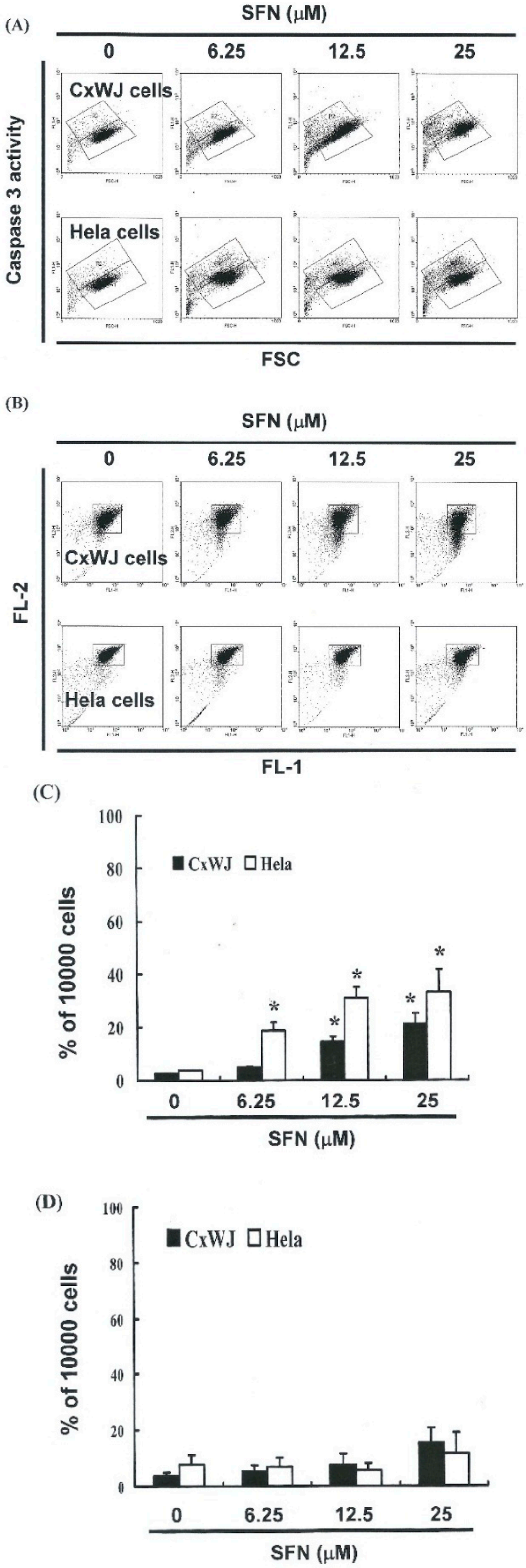

Figure S2. (**A**) Caspase-3 activity and (**B**) JC-1 in cervical cancer cell lines following 24 h of SFN treatment (0, 6.25, 12.5, and 25 μM). Following treatment, cells were harvested and labeled using FITC rabbit anti-active caspase-3 or JC-1. Activation was quantified using flow cytometry; (**C**) Quantification of caspase-3 activity and (**D**) quantification of JC-1. All data is reported as the mean (±SEM) of at least three separate experiments. Statistical analysis was performed using a *t*-test, with differences considered significant at a level of * $p < 0.05$ versus the 0 μM control group.

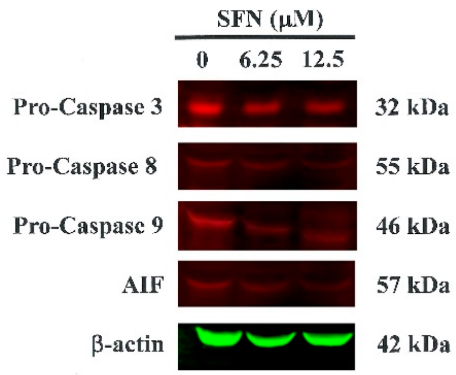

Figure S3. Influence of SFN on the expression of caspase-3, caspase-8, caspase-9 and AIF proteins in HeLa cells. Cells were treated with SFN (0, 6.25 and 12.5 μM) for 24 h, and proteins were subsequently detected by western blotting representative blot from 3 independent experiments.
